# Supplementary material for: Constitutive, Basal, and β-Alanine-Mediated Activation of the Human Mas-Related G Protein-Coupled Receptor D Induces Release of the Inflammatory Cytokine IL-6 and Is Dependent on NF-κB Signaling
Source: Int J Mol Sci. 2021 Dec 9;22(24):13254. doi: 10.3390/ijms222413254 (PMC8703779; doi:10.3390/ijms222413254)
Supplement: Supplementary file 1 [file ijms-22-13254-s001.zip › ijms-1044970-SI.pdf]

International Journal of
Molecular Sciences

Supplementary Material

1.1 β-Alanine-Mediated Activation of MRGPRD Induces Release of IL-6 from HT1080 cells

HT1080 cells were cultured in 100 mm dishes using DMEM supplemented with 10% FBS and 1% penicillin-streptomycin (incubator setting, 37°C with 5% CO_2). HT1080 cells were collected by trypsinization and 2.5 × 10^5 cells/well were seeded in a 6 well cell culture plate. After 16 h, medium was removed, and cells were washed once with 1 ml phosphate-buffered saline (PBS). Cells were transfected with 2 µg plasmid cDNA (i.e. pCMV-GS or pCMV-MRGPRD-HA) using Lipofectamine 2000 as per the manufacturer's instructions. After 4 h, transfection medium was replaced with 2 mL DMEM supplemented with 10% FBS and 1% penicillin-streptomycin and the plate was returned to the incubator. 24 h later, medium was removed and replaced with 2 mL DMEM to incubate the cells overnight in medium without serum (starvation step). Next day, after 24 h, i.e., 48 h from transfection, cells were washed once with 1 ml PBS and 2 mL DMEM was added to each well containing either vehicle control (percentage of milli-Q was 0.2%) or β-alanine (dissolved in milli-Q; 0.2%). The final concentration of β-alanine was 100 µM. After treatment, the plate was returned to the incubator for another 24 h incubation. Subsequently, i.e., 72 h post-transfection, cell supernatant medium was collected in a 2 ml microcentrifuge tube and spun at 1000 *g* for 5 min at 4 °C to pellet down debris. The supernatant medium was transferred to a fresh microcentrifuge tube and stored at −80°C until assayed by ELISA. For IL-6 assessment the collected supernatant medium was thawed on ice and sample was diluted (1:100 and 1:10) in assay diluent buffer and IL-6 estimation was performed using an IL-6 ELISA (R&D Systems; Cat# D6050) kit as per the manufacturer's instructions. The calculated IL-6 concentrations from collected supernatant medium were represented as ng/mL (Figure S1).

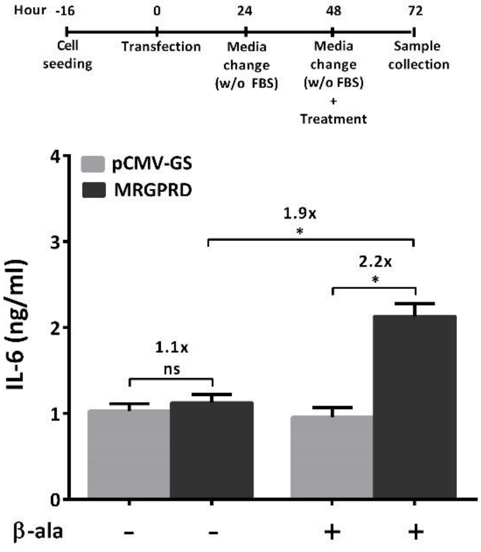

Figure S1. Activation of MRGPRD mediates IL-6 release in HT1080 cells. HT1080 cells transiently expressing empty vector (pCMV-GS) or MRGPRD-HA receptor were stimulated with either β-alanine (100 µM; final concentration/well) or vehicle control. A significant, 1.9-fold higher release of IL-6 was observed from MRGPRD-expressing cells treated with β-alanine as compared to vehicle-treated. Also, IL-6 levels of β-alanine treated MRGPRD-expressing cells were nearly 2.2-fold higher than those of empty vector expressing control cells. The IL-6 release is represented in ng/ml (average of n = 2 experiments). All values are represented as the mean ± s.e.m. Statistical significance was determined using one-way analysis of variance (ANOVA) and Sidak's post hoc test was applied for multiple comparisons. * $P \leq 0.05$ was considered significant and 'ns' is non-significant.

1.2 β-Alanine-Mediated Activation of MRGPRD Induces IP1 Accumulation in HeLa cells

HeLa (mock) cells and HeLa cells stably expressing MRGPRD-NLuc (with 1000 µg/ml Geneticin) were cultured in 100 mm dishes using DMEM supplemented with 10% FBS and 1% penicillin-streptomycin (37 °C with 5% CO_2). The cells were collected by trypsization and 2 × 10^4 cells/well were seeded in 96 white, flat, opaque bottom well plate (CELLSTAR; Cat# 655083). 24 h post seeding, the medium was removed and replaced with 100 µL DMEM for overnight starvation. The next day, i.e, 48 h from seeding, the medium was replaced with 100 µL stimulation buffer (without β-alanine) containing either vehicle control (final concentration of DMSO was 0.1%) or desired concentration of inhibitor (dissolved in DMSO; 0.1%), and plate was kept in incubator for 1 h. Post-incubation, cells were stimulated with 40 µL stimulation buffer containing vehicle control (DMSO; 0.1%) or inhibitor (dissolved in DMSO; 0.1%) with vehicle control (percentage of milli-Q was 0.1%) or β-alanine (dissolved in milli-Q; 0.1%). The final concentration of β-alanine was 1 mM/well. Plate was then returned to the incubator (37 °C with 5% CO_2) for 1 h. Afterwards 30 µL of donor (IP1- cryptate antibody) and 30 µL acceptor (IP1-d2) was added to wells as per the manufacturer's instructions (Cisbio; IP-One-Gq HTRF assay). The plate was incubated at 25 °C for 1 h, subsequently read using an EnVision multimode plate reader. Using standard curve, HTRF ratios were converted into IP1 concentration in nM. The relative fold change (ΔF) was obtained by normalizing IP1 to that of its respective control. The fold-change (ΔF) was further normalized to the ΔF of β-alanine-stimulated MRGPRD-expressing cells (i.e., ΔFmax) and expressed as percentage.

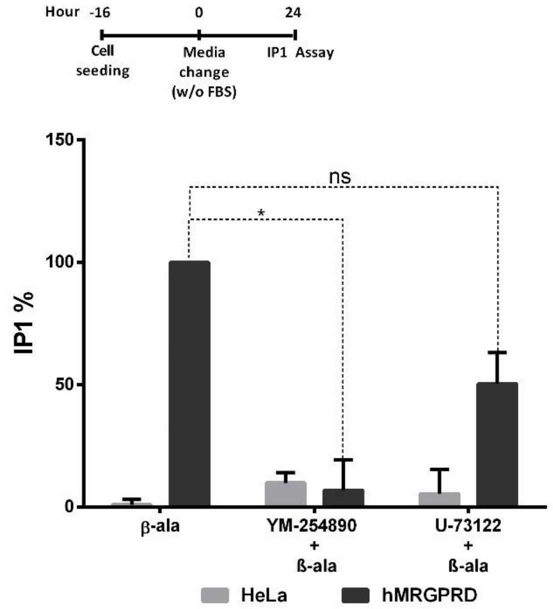

Figure S2. Activation of MRGPRD increased inositol monophosphate (IP1) accumulation in HeLa cells. HeLa (mock) and Hela cells stably expressing MRGPRD-NLuc were pre-treated with either vehicle control or the Gαq inhibitor (YM-254890; 10 µM) or the pan-PLC inhibitor (U-73122; 10 µM) for 1 h before being stimulated with β-alanine (1mM). β-alanine-stimulated MRGPRD-expressing HeLa cells induced IP1 accumulation. No induction was observed in cells pre-treated with Gαq inhibitor, whereas partial reduction was observed for pan-PLC inhibitor. The IP1 accumulation is represented in percentage of ΔF/Fmax (average of n = 2 experiments). All values are represented as the mean ± s.e.m. Statistical significance was determined using one-way analysis of variance (ANOVA) and Sidak's post hoc test was applied for multiple comparisons. * $P \leq 0.05$ was considered significant and 'ns' is non-significant.
